# A Concise History of the Principal Discoveries in Anatomy

**Published:** 1799-11

**Authors:** 


### *A Concife Hiftory of the Principal Difcoveries in Anatomy.*

[ Continued from our laft Number, pp. 281—282. ]

§. 19. Six years had elapfed after the publication of Serveto's work, mentioned in the preceding number, when Columbus, with great oftentation, according to his ufual method, maintained that the doctrine of the fmaller circulation of the blood through the lungs was his own difcovery. We cannot, however, deny him the merit of having given a more diftinct account of this important fubject than Serveto himfelf, who merely fpeaks of pure blood, while Columbus maintains that the blood, mixed with animal fpirits, returns from the lungs. Aranzi, fpeaking on this fubject, candidly owns the difficulties which have induced him to difagree with with COLUMBUS, and yet to hesitate in embracing the opinion, that the blood could exude or penetrate through the solid septum, from the right to the left ventricle of the heart. And if it should be granted that such an exudation were possible, or even really took place, he could not conceive why the blood did not *return* also to the right ventricle of the heart, through the same holes or pores hypothetically admitted; and thus perhaps disturb the order of nature. It was also inexplicable to him, what purpose the coronary veins, and still more the great pulmonary artery, could answer, if the blood was merely to penetrate through the septum from one ventricle into the other: nor could he comprehend why the pulmonary artery should be so capacious and extensive, if it were designed to convey only the air from the lungs, and not to conduct, at the same time, the blood to the heart. Nay, we even frequently find, after death, the pulmonary artery quite full of blood; and also the valves on the pulmonary veins and arteries. In short, it is obvious, that ARANZI is doubtful, which side of the question he ought to embrace; and he concludes with the common-place remark, "that many things happen in this sublunary world, of which philosophers could scarcely dream." VAROLI was also acquainted with the relation subsisting between the pulmonary artery and the pulmonary vein; but he does not enter upon a distinct explanation of the functions of these vessels.

ANDR. CAESALPINUS, of Arezzo, first physician to the Pope, was the next author who wrote a very detailed treatise on the passage of the blood through the lungs. He acquired very great reputation, by the original manner in which he illustrated the principles of the peripatetic philosophy, and by the contest he maintained on this subject with TAURELLUS.* CAESALPINUS proceeds on the principle, that the heart is not refrigerated during respiration; for, in cold water, the heart of animals is very soon deprived of its vital power, which it retains much longer in warm water; and, according to him, the lungs merely serve to cool the heated blood. This blood flows from the right ventricle of the heart into the pulmonary artery, and from this, by means of numerous anastomoses, it returns through the pulmonary vein to the left ventricle of the heart. The branches of the trachea run collateral with the ramifications of the pulmonary veins: the former do not form anastomoses with the latter, but merely serve, by the contact with atmospheric

---

* Compare the work entitled, "*Memorie degli uomini di Toscana,*" vol. i. p. 93; also BAYLE, vol. ii. p. 118.—BRUCKER, vol. iv. p. 220; and NICERON *Memoires*, vol. xlii. p. 164.

air, to cool the sides of the vessels containing venous blood, and consequently that fluid itself. He farther asserts, that it is absurd to call the pulmonary artery *vena arteriosa,* merely because it, like the *vena cava,* is situated near the right ventricle of the heart; it is nevertheless a true artery, and bears the greatest resemblance to the *aorta:* the name of *arteria venosa,* which has been given to the pulmonary vein, is equally inconsistent; as this vessel, notwithstanding its termination in the left ventricle of the heart, possesses all the properties of a vein.

Although CAESALPINUS had such accurate ideas on the smaller circulation, he admits the exudation of the blood through the septum cordis.* This passage, however (in the opinion of Prof. SPRENGEL), very clearly demonstrates, that this learned physician was perfectly acquainted with the circulation of the blood through the lungs. CAESALPINUS *likewise conceived the great circulation of the fluids through the whole body,* the knowledge of which he certainly could not acquire from the works of either FABRICIUS or HARVEY; for we find remarkable proofs of this assertion in his other works. He first remarked the swelling of the veins between their terminations, and an applied ligature; whence he concluded, that the opinion commonly received, with respect to a progressive motion in the veins, rested upon an erroneous conjecture. But in the same passage he still indulges in speculations, which led him to compare the circulation of the blood through the veins to the flux and reflux of the sea; and not being acquainted with the valves which must necessarily obstruct the *retrograde* course of the blood through the veins, he does not appear to have had clear and fixed ideas on the subject.† In another work, however, he treats of the mere reflux of the blood through the veins, with such precision, that Prof. SPRENGEL would not hesitate a moment to consider him as the discoverer of the great circulation, if he were more consistent in his assertions, and if he had set out with the discovery of the valves in the veins. The following passage, which occurs in the work of CAESALPINUS "*De Plantis,*" 4to, printed at Florence in 1583, is submitted to the consideration of the reader, whether, and how far, this author is entitled to that honour: "*Qua autem ratione fiat* "*alimenti attract nutritio in plantis, consideremus. Nam in animalibus* "*videmus alimentum per venas duci ad cor, tanquam ad officinam caloris* "*insiti, et adepta inibi ultima perfectione, per arterias in universum corpus* "*distribui, agente spiritu, qui ex eodem alimento in corde gignitur.*" Lib. I. c. 2.

* CAESALPINI *Quaestiones Peripateticae.* lib. v. c. 4. p. 528. (Folio, Lugdun. 1588.)

† CAESALPINI *Quaestiones Medicae,* lib. ii. cap. xvii. f. 234. (Quarto, Venet. 1593.

c. 2. p. 3.—Prof. SPRENGEL candidly adds, that his veneration for the immortal HARVEY is too great to harbour the moſt diſtant ſuſpicion, that this illuſtrious character would have condeſcended to uſurp the honour of a diſcovery, if he had known, that an earlier inquirer had already anticipated the merit. It is, however, ſurpriſing that FABRICIUS,* the excellent anatomiſt, had not more perſpicuous ideas on the functions of the pulmonary veſſels, and that he was ſo invariably attached to the eſtabliſhed errors and prejudices of his predeceſſors.

§ 20. The circulation of the blood in the human fœtus was ſo thoroughly and ſucceſsfully examined during the ſixteenth century, that the *foramen ovale* between the two auricles of the heart was ſoon diſcovered by anatomiſts: they alſo obſerved that this foramen in the embryo leads from the cyſt of the vena cava to the cyſt of the pulmonary vein, and that it is cloſed by a valve which prevents the reflux of the blood; but that, in adults, it forms a cavity which is ſurrounded by an iſthmus, and which is almoſt completely impermeable. This courſe of the blood in the fœtus had already been noticed by GALEN;† and the paſſage below alluded to is ſo concluſive, and at the ſame time proves that this ancient writer poſſeſſed ſo correct a knowledge of the ſtructure of the infantile body in the womb, that we cannot withhold from him our admiration. But GALEN had made ſtill greater progreſs; he was acquainted with the arterial canal that receives and immediately conducts to the aorta the blood which proceeds from the head as well as the upper extremities of the child, and which cannot paſs through the umbilical veins, nor through the foramen ovale, but which flows through the aſcending vena cava into the ſinus cordis dexter. GALEN was no ſtranger to this particular branch of the pulmonary artery; but he did not clearly underſtand its deſtination.

After the time of GALEN, FALLOPIUS was the firſt author who gave a diſtinct deſcription of it; but he certainly betrayed his want of attention, by aſſerting that this branch proceeded as far as the ventricle of the heart; nor was he correct when he maintained that the blood from the aorta flowed through this channel, into the pulmonary artery, and into the heart, as it takes the direct contrary courſe.‡ VESALIUS himſelf had at firſt no knowledge of either the foramen ovale, or the arterial canal, and conſequently does not mention them in his larger work. But after FRANC. ROTA had intimated to him in a friendly epiſtle,

* See his work "*De Reſpiratione*," cap. xii. p. 184.

† *De Uſu Part.* lib. xvi. p. 533.

epiftle, that the defcription of thefe parts of the embryo which were fo well known to GALEN, is reluctantly omitted in his claffical work, the attention of VESALIUS was thus roufed: he afterwards mo e carefully examined thefe parts, difcovered the valve in the foramen ovale, as well as the arterial canal,* and regrets in the paffage here referred to, that he had not fooner applied himfelf to this important inquiry.

The next anatomical writer was ARANZI, who publifhed a detailed and authentic hiftory of the foramen ovale, of its valve, of the concretion of the former which takes place after birth, of the arterial canal, and its ligamentous ftructure in adults. But he likewife erred, as well as FALLOPIUS, in maintaining that the blood is conducted through this canal from the aorta to the lungs and the heart.

After thefe celebrated men had illuftrated this fubject, in a manner reflecting credit on the age in which they lived, there appeared a pfeudo-difcoverer of the name of BOTALLI, a pupil of the great FALLOPIUS, and who arrogantly appropriated to himfelf the refpective difcoveries of the foramen ovale, and the arterial canal: nay, fome authors have, either from falfe liberality or ignorance, called thefe parts after his name.

VAROLI defcribes the foramen ovale and the arterial canal, in a manner exactly fimilar to that of his predeceffors, and commits the fame error with refpect to the deftination of the latter.† CARCANI adds no original remarks to the defcription of thefe parts, except the obfervation, that the arterial canal in the embryo is two fingers breadth from the bafis of the heart; while in adults it is four.‡ ALBERTI,§ ULMUS,|| and DU LAURENS¶ made no improvements upon the previous defcription given by FALLOPIUS. It is to FABRICIUS we are indebted for the firft drawings of thefe parts, which, with the exception of the fixth plate, fig. 15, E. F. reprefenting the arterial canal, are faithful copies of nature.

The anatomifts of this century beftowed confiderable attention on the courfe of the venous duct, which empties itfelf from the umbilical vein into the vena cava, or into the hepatic vein. VESALIUS difcovered this duct, and ftated it to be only half the fize of the umbilical vein from which it takes its origin.** Soon after it was difcovered, EUSTACHIUS

* *Vefal. Exam. Obfervat. Falloft.* p. 798.

† *Varoli Anatomia*, lib. iv. c. 5. p. 108.

‡ *Carcani Anatomia*, p. 14. s. 28. 34. s.

§ *Alberti Hiftoria Anatomia*, p. 155.

|| *De Liene*, f. 21. 6. (Quarto, 1578.)

¶ *Laurent. Hiftor. Anatom.* lib. viii. Quæft. 27. p. 684.

** *Vefal. Exam. Obfervat.* FALLOPII, p. 798.

CHIUS caused an accurate representation of it to be published.* ARANZI observed a double duct of this kind, one leading to the vena portæ, the other to the vena cava; the latter has been denominated after him the venous duct of ARANZI.† FABRICIUS has furnished a more distinct delineation of this canal than his rival EUSTACHIUS.‡

[ To be continued in our next Number. ]

* *Eustach. Tab.* xvii. fig. 1. (w.)

† *Arant de human. foetu.* c. 14. p. 40.

‡ *De format foetu,* Tab. vii. fig. 16. (j.) Tab. viii. fig. 17. (c.)

